# Supplementary material for: Accelerating network layouts using graph neural networks
Source: Nat Commun. 2023 Mar 21;14:1560. doi: 10.1038/s41467-023-37189-2 (PMC10030870; doi:10.1038/s41467-023-37189-2)
Supplement: Supplementary file 1 — Supplementary Information [file 41467_2023_37189_MOESM1_ESM.pdf]

Supplementary information

Accelerating Graph Layouts Using Graph Neural Networks

Csaba Both[1], Nima Dehmamy[2], Rose Yu[3], Albert-László Barabási[1,4,5*]

[1] Network Science Institute, Northeastern University, Boston, MA, USA, [2] MIT-IBM Watson AI Lab, IBM Research, Cambridge, MA, USA, [3] Department of Computer Science and Engineering, University of California, San Diego, CA, USA, [4] Department of Medicine, Brigham and Women's Hospital, Harvard Medical School, Boston, MA, USA [5] Department of Data and Network Science, Central European University, Hungary

(*Corresponding author: barabasi@gmail.com)

Contents

A. Neural network architecture for graph layout 2

A.1. NodeMLP 2

A.2. NeuLay 2

B. Computational Complexity 3

C. Convergence Rate 5

D. Training and the setup of experiments 8

D.1. Hidden Layers 8

D.2. Hybrid Optimization 8

D.3. Convergence and Stopping Criteria 9

E. Experiments 11

A. Neural network architecture for graph layout

Neural networks have been successfully reparametrized an optimization variable in topology optimization [1], and helped encode the state of a system in deep reinforcement learning [2]. Here we describe the details of the parameters and structure of the neural network models we purposed in this work.

A.1. NodeMLP

In NodeMLP (Fig. 1 b), we start from random node embedding $Z \in \mathbb{R}^{N\times m}$ and project it down to a d dimensional space using a fully-connected (FC) network layer ($m > d$). The output is $X = \sigma(ZW + b)$, with weight matrix $W \in \mathbb{R}^{m\times d}$, bias vector b (length d), and activation function σ. In NodeMLP, the trainable parameters are $\theta = \{Z, W, b\}$. Even though NodeMLP involves many more parameters than FDL, it converges significantly faster (Fig. 1 e, f) to similar energies (Fig. S1). In NodeMLP the energy is $\mathcal{L}(X(\theta))$, a function of the high-dimensional parameter space defined by components of $\theta = \{Z, W, b\}$. The fact that NodeMLP converges faster suggests that NodeMLP may be finding more efficient routes for gradient descent in the high dimensional energy landscape than FDL.

A.2. NeuLay

NodeMLP does not exploit the graph structure to parametrize $X(\theta)$. It means that the graph adjacency matrix A is not part of the $X(\theta)$ function. To leverage the higher order structural information in the graph, we introduce NeuLay (Fig. 1 c). NeuLay uses Graph Convolutional Networks (GCN) [3]:

$$G(X) = \sigma(f(A)XW) \tag{1}$$

where $f(A)$ is the aggregation function and W is trainable parameter. GCN extracts relational information from graphs by aggregating each node information i.e., node positions

from its neighbors. We find that including GCN layers in $X(\theta)$ yields one to two order of magnitude speed-up in FDL (Fig. 1 e, f).

Specifically, NeuLay starts with a high-dimensional embedding $Z \in \mathbb{R}^{N\times m}$. We use a two-layer GCN, NeuLay-2, with *residual connections* (i.e. concatenating the output of each layer to the final output layer) to extract higher-order structures. Also, the residual connection layer help to solve the graph isomorphism problem that appears when we lay out topologically the same nodes e.g. when three nods have the same topological relationship, forming a triangle (Fig. S2). The first layer takes Z as input and produces the output $G_1 = \sigma\left(f(A)ZW^{(1)}\right)$, which is passed to the second layer which outputs $G_2 = \sigma\left(f(A)G_1W^{(2)}\right)$. Here $W^{(1)} \in \mathbb{R}^{m\times h_1}$ and $W^{(2)} \in \mathbb{R}^{h_1\times h_2}$ the weight matrix of the first and second GCN, and σ is an activation function. The first GCN layer embeds each node as an h_1 dimensional vector while aggregating features from the neighboring nodes using an aggregation function $f(A)$. Similarly the second GCN layer embeds each node as an h_2 dimensional vector. We use the symmetrized degree normalized adjacency matrix as the aggregation function $f(A) = \tilde{D}^{-1/2}\tilde{A}\tilde{D}^{-1/2}$, where $\tilde{A} = A + I$ and $\tilde{D}_{ii} = \sum_j \tilde{A}_{ij}$ is the degree matrix of $\tilde{A}$. Finally, we pass the high dimensional embedding Z along with the two-layer GCN outputs, G_1 and G_2, to the final FC layer with linear activation $\sigma(x) = x$. We concatenate the outputs of all three layers along the feature dimension (columns) into $G_3 = [Z|G_1|G_2] \in \mathbb{R}^{N\times(m+h_1+h_2)}$. We then pass G_3 to an FC layer to project down to the d dimensional $X \in \mathbb{R}^{N\times d}$ as layout positions

$$X = G_3W + b = ZW_Z + G_1W_1 + G_2W_2 + b \tag{2}$$

Here $W = [W_Z|W_1|W_2] \in \mathbb{R}^{(m+h_1+h_2)\times d}$ is the weight matrix of the FC layer.

B. Computational Complexity

The elastic energy is calculated for only linked node pairs, where the adjacency element is not zero, but the repulsive term is calculated for each node pair. Computationally this is a $\mathcal{O}(N^2 + M)$ problem where N is the number of nodes and M is the number of links.

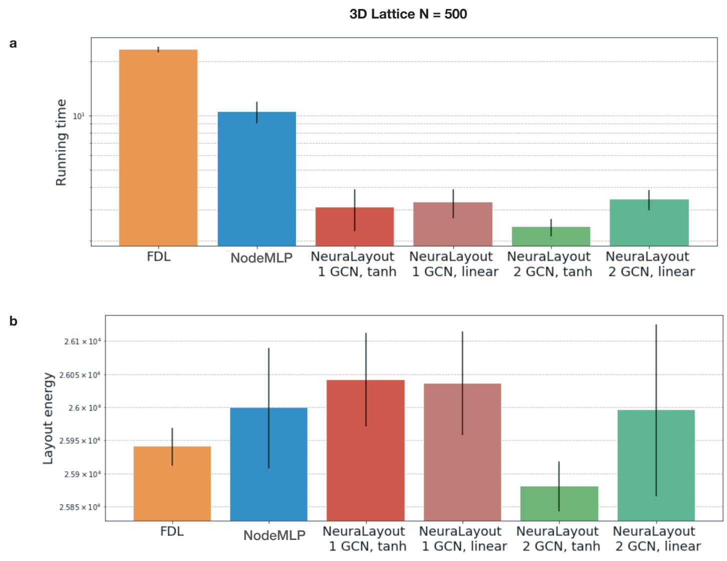

FIG. S1: **3D Lattice.** Comaprison of NeuLay with linear and non-linear activations.

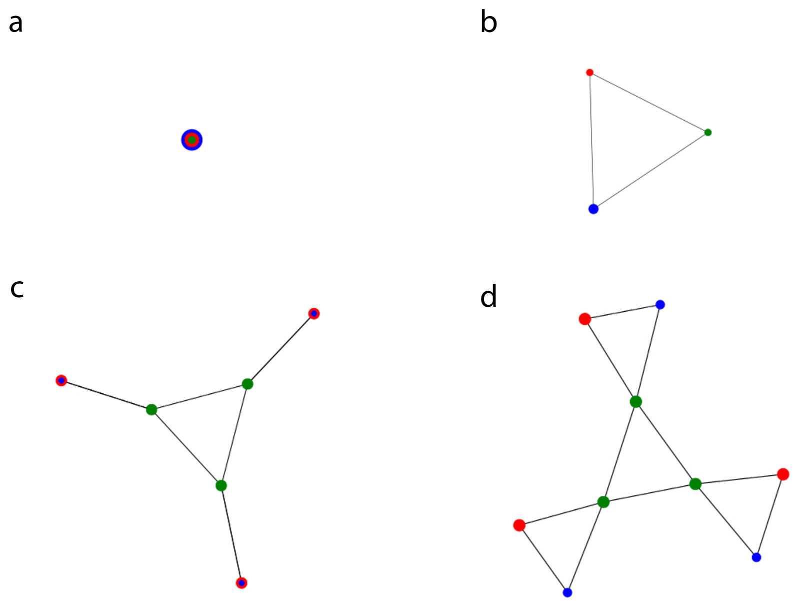

FIG. S2: **Graph Isomorphism.** Symmetric graphs by (a,c) GCN and (b,d) ResGCN model. Two simple examples for the graph isomorphism problem e.q. in the triangle, all three nodes are topologically equivalent. Hence this fact, the GCN model cannot distinguish these nodes from each other and forces them together.

The exponential repulsion term is generally negligible at distances larger than $|r_{ij}| > 4r_0$ and mostly relevant locally for short distances. For determining efficiently the relevant node pairs we use a k-d tree [4] method. The k-d tree computational complexity is $\mathcal{O}(NlogN)$

instead of $\mathcal{O}(N^2)$ in each iteration step, it is more efficient if we apply a scheduler and use it in certain steps [5].

To ensure a fair comparison with state-of-the-art methods, we also use k-d trees in the FDL part of our hybrid optimization. We use a scheduler to rebuild the tree only in certain steps [5]. In our simulations we rebuild the tree in every five step. Thus, we ensure that the observed speedup is indeed due to NeuLay.

C. Convergence Rate

Our empirical results lead to the hypothesis that top outlier eigenvectors in the graph drive the speedup achieved by NeuLay. To analytically derive the role of these outliers, we analyzed the equations for the rate of change of the loss $d\mathcal{L}/dt$.

In NeuLay node positions $X(\theta)$ are functions of the neural network parameters θ which include $Z, W^{(1)}, W^{(2)}, W, b$. The GD updates in eq. 1 (Main text) becomes GD equations for θ

$$\frac{d\theta_i}{dt} = -\varepsilon \frac{\partial \mathcal{L}}{\partial \theta_i} \tag{1}$$

Using this we can write

$$\frac{d\mathcal{L}}{dt} = \sum_i \frac{d\theta_i}{dt} \cdot \frac{\partial \mathcal{L}}{\partial \theta_i} = -\varepsilon \sum_i \left\| \frac{\partial \mathcal{L}}{\partial \theta_i} \right\|^2 = -\varepsilon \|\nabla \mathcal{L}\|^2 \tag{2}$$

NeuLay works the best in the early stages of the optimization. Early on, the nodes are initialized far enough to give them space to move past each other. However, since node positions are random, many links are very stretched. Hence, in early stages, V_{NN} is relatively small and the dynamics is dominated by elastic forces from V_{el}. For simplicity, let all layer activation functions be linear, meaning $\sigma(x) = x$, and put biases to zero $b = 0$. With these

assumptions, for NodeMLP we have

$$
\begin{aligned}
X &= ZW \\
\mathcal{L}_{DF} &= \frac{1}{2}\mathrm{Tr}\left[X^T L X\right] = \frac{1}{2}\mathrm{Tr}\left[W^T Z^T L Z W\right] \\
\frac{d\mathcal{L}_{DF}}{dt} &= -\varepsilon(\left\|LZWW^T\right\|^2 + \left\|Z^T LZW\right\|^2)
\end{aligned} \tag{3}
$$

Doing the same for NeuLay with one GCN layer yields

$$
\begin{aligned}
X &= G_1 W = f(A)ZW^{(1)}W, \quad W_2 \equiv W^{(1)}W, \quad L_3 \equiv f(A)^T L f(A) \\
\mathcal{L}_{NL} &= \frac{1}{2}\mathrm{Tr}\left[W_2^T Z^T L_3 Z W_2\right] \\
\frac{d\mathcal{L}_{NL}}{dt} &= -\varepsilon(\left\|L_3 Z W_2 W_2^T\right\|^2 + \left\|Z^T L_3 Z W_2\right\|^2)
\end{aligned} \tag{4}
$$

where the gradients with respect to W and $W^{(1)}$ are summarized as gradients for W_2. We may now compare the early stage evolution of the loss in NodeMLP (3) and NeuLay (4). Since W and W_2 both represent trainable $m \times d$ matrices, we can equate them in the two equations. Hence, the key difference is that L in (3) is replaced by $L_3 = f(A)^T L f(A)$ in (4). This leads to top eigenvectors of $f(A)$ getting larger weights in NeuLay. To show this, we use the spectral expansion $f(A) = \sum_i \lambda_i \psi_i \psi_i^T$. Since A is undirected in FDL $f(A) = D^{-1/2} A D^{-1/2}$ is Hermitian and ψ_i form a complete basis. The difference between (3) and (4) is that replacing $L \to L_3$ results in $Z \to f(A)Z$. Expanding Z in ψ_i we have

$$
Z = \sum_i \psi_i \psi_i^T Z = \sum_i \psi_i z_i, \qquad z_i = \psi_i^T Z \in \mathbb{R}^m \tag{5}
$$

$$
f(A)Z = \sum_i \lambda_i \psi_i z_i \qquad f(A)^p Z = \sum_i \lambda_i^p \psi_i z_i \tag{6}
$$

The extra λ_i factor in $f(A)Z$ means the overlap with ψ_i with larger eigenvalues is magnified. For 2 layers of GCN we have $f(A)(f(A)ZW^{(1)})W^{(2)} = f(A)^2 Z W_2$, resulting in a factor of λ_i^2 in the expansion. Thus, if the spectrum of $f(A)$ has a set of outliers such that $\forall j \in out, \lambda_j \gg \mathrm{mean}_i[\lambda_i]$, the ψ_j will dominate the expansions (6) $f(A)^p Z \approx \sum_{i \in out} \lambda_i^p \psi_i z_i$.

This is assuming the overlaps z_i are not much smaller for outliers than the rest. This is a reasonable assumption as Z is initialized at random and thus its overlap z_i with any basis vector is of the same order of magnitude.

To see how this enhancement of outliers in $f(A)^p Z$ affects $d\mathcal{L}/dt$ we will consider the specific example of SBM. Consider an SBM with two blocks and with the same average degree $\langle k\rangle$ in both blocks. The degree matrix is approximately $D \approx \langle k\rangle I$. This leads to all of A $f(A) = D^{-1/2}AD^{-1/2}$ and $L = D - A$ having the same eigenvectors.

$$\text{Uniform SBM: } A = \sum_i \lambda_i \psi_i \psi_i^T, \quad f(A) = \frac{1}{\langle k\rangle}\sum_i \lambda_i \psi_i \psi_i^T, \quad L = \sum_i \left(\langle k\rangle - \lambda_i\right)\psi_i \psi_i^T \tag{7}$$

Using this and equating $W_2 = W$, we can write the losses (3) and (4) as

$$L_3 = f(A)^k L f(A)^k$$
$$\mathcal{L}_{DF} = \frac{1}{2}\mathrm{Tr}\left[W^T Z^T L Z W\right] = \frac{1}{2}\sum_i \left(\langle k\rangle - \lambda_i\right)\|z_i W\|^2 \tag{8}$$
$$\mathcal{L}_{NL} = \frac{1}{2}\mathrm{Tr}\left[W^T Z^T L_3 Z W\right] = \sum_i \lambda_i^{2p}\left(\langle k\rangle - \lambda_i\right)\|z_i W\|^2 \tag{9}$$

Taking the gradients, we have

$$\frac{d\mathcal{L}}{dt} = -\varepsilon\left\|\frac{\partial\mathcal{L}}{Z}\right\|^2 - \varepsilon\left\|\frac{\partial\mathcal{L}}{W}\right\|^2$$
$$\frac{d\mathcal{L}_{DF}}{dt} = -\varepsilon\sum_i \left(\langle k\rangle - \lambda_i\right)^2\|z_i W W^T\|^2 - \varepsilon\left(\sum_i \left(\langle k\rangle - \lambda_i\right)\|z_i^T z_i W\|^2\right)^2$$
$$\frac{d\mathcal{L}_{NL}}{dt} = -\varepsilon\sum_i \lambda_i^{2p}\left(\langle k\rangle - \lambda_i\right)^2\|z_i W W^T\|^2 - \varepsilon\left(\sum_i \lambda_i^{p}\left(\langle k\rangle - \lambda_i\right)\|z_i^T z_i W\|^2\right)^2 \tag{10}$$

As we see, the rate of change of each mode ψ_i has a $\langle k\rangle - \lambda_i$ factor. This means that the leading eigenvectors have the slowest evolution in the NodeMLP loss $\mathcal{L}_{DF}$. But in NeuLay, the gradients for mode ψ_i are multiplied by λ_i^p, which means that the rate of gradient descent dz_i/dt is increased by a factor of λ_i^p. As a result, the rate of decrease in loss $d\mathcal{L}_{NL}/dt$ also sees an increase due to the λ_i^p factors.

In conclusion, we find that the GCN layers magnify the rate of convergence of the outlier eigenvectors by a factor λ_i^p for p GCN layers. This compensates for the slow convergence rate of these eigenvectors, which was proportional to $\langle k \rangle - \lambda_i$ in NodeMLP and FDL.

D. Training and the setup of experiments

D.1. Hidden Layers

Using wide GCN layers (i.e. large h_1, h_2) is computationally expensive and after a certain point, it is not beneficial. We explored the hyperparameter space to determine the best configuration of the hidden dimensions. The results show that if the third hidden dimension is equal to the final layout dimension the NeuLay achieves the best result in time and energy. For the first two hidden dimensions, we did not find any clear patterns that guarantee a good performance. According to our experiments, the first two layers have to be much larger than the third one, but we have to be careful with the width of the layers especially in large networks, as it could be computationally expensive. For all large networks we used 100 hidden dimensions for both GCN layers.

D.2. Hybrid Optimization

When, we optimize the network layout initial configuration, the node positions are randomly generated in $N^{1/3}$ range until we find the lowest energy layout. We adopt a hybrid strategy to optimize the total energy and to update the parameters in Eq. 3 (Main text). In our experiments, we find that NeuLay approaches good layouts much faster than FDL and NodeMLP. In the final stages of optimization, however, we find that the NeuLay takes a long time to fine-tune the energy. Hence, we propose to first optimize the total energy using NeuLay until the energy almost plateaus (Fig. S3). We then remove the GCN layers and optimize only the d dimensional positions (i.e. fine-tune using FDL). This hybrid optimization approach converges considerably faster than either NeuLay or FDL alone.

We implemented our code by using **PyTorch** and for the experiments we used **RMSProp**

optimizer with learning rate 0.01.

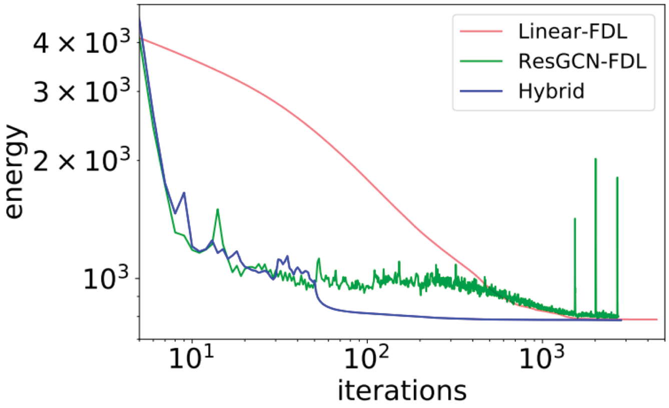

FIG. S3: **Hybrid optimization.** Loss energy curves of ResGCN-FDL (only using GCN model, green), Linear-FDL (FDL, red) algorithm, and Hybrid (GCN until the loss curve starts converging to a plateau, then we switch to FDL) for laying out the flavor network.

As we have seen the NeuLay model (GCN) can benefit from the graph structure and especially from the communities. Figure S4 shows that if we fix the node and edge number in a graph and change the network topology, the additional communities help the convergence in the NeuLay model (black curve) compared to the FDL model (red curve). Until 100 steps the NeuLay model clearly provides a faster way to the energy minimum. However, after the cross-section of the inflection point of the energy curve of FDL, it could be more advantageous to use the FDL. This is significant proof of why and when the NeuLay could be beneficial in the energy-based graph drawing problem.

D.3. Convergence and Stopping Criteria

To measure the speedup of different methods we use the same stopping criteria for both the FDL and the NeuLay, which $10^{-9} \times \sqrt{N}$ change of the loss function over 10 patience steps, where N is the number of nodes in the network. The optimization stops if the loss changes less than a fixed threshold value over the patience window. To change from NeuLay we use a softer stopping criteria with a lower patience value, $10^{-4} \times \sqrt{N}$.

However, for large grid-like networks like RGG, given the rough energy landscape twisting only one part of the layout is sufficient to get trapped in a different local minima. NeuLay

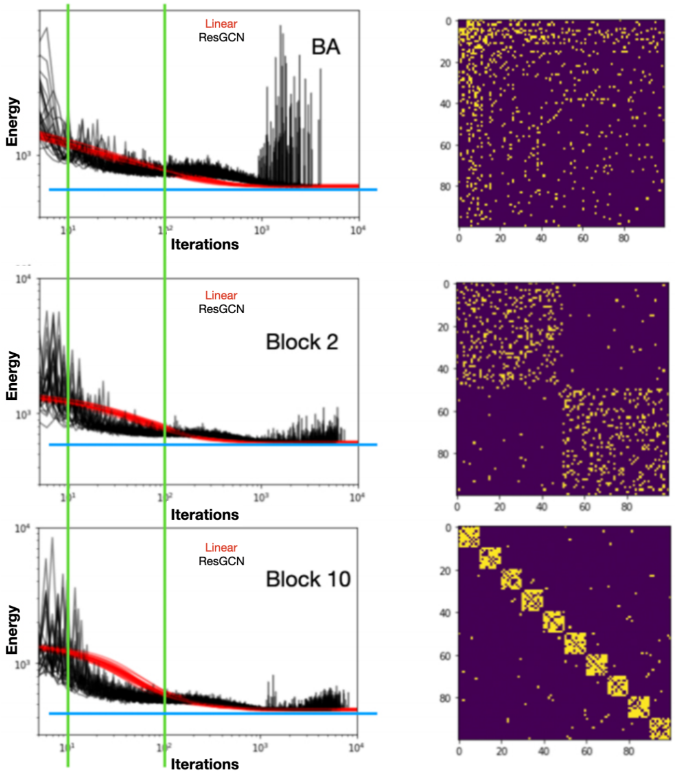

FIG. S4: **Stochastic Block Model** Loss energy curves of NeuLay-2 (ResGCN - two GCN layers with residual connections, black) and of FDL (Linear, red) method for laying out graphs with a fixed number of nodes and edges but with different topology (adjacency matrices in the right column). All three examples (Barabasi-Albert model graph, Stochastic Block Model with 2 and 10 blocks) show that at least until 100 steps the NeuLay-2 model has a deeper energy state compare to the FDL. Furthermore, the number of blocks influence the depth of this early energy drop, if the graph contains more blocks the energy gradient is larger.

consistently reaches a deeper energy state for these networks. In such cases we used the FDL energy as a stopping criteria for the NeuLay measure the speedup properly. For other network ER or BA we do not have this problem.

E. Experiments

To demonstrate the value of NeuLay-2, we tested it on real networks. NeuLay-2 can dramatically accelerate the convergence in the early stages of the optimization (Fig. S5 a, Fig. S6). NeuLay-2 is not only faster but also converges to a deeper energy state. The energy surface of large networks contains enormous number of local minima. Hence, it is difficult to easily spot the differences between two layouts, particularly if they are trapped into two local minima close to each other (Fig. S5 b); however, in other networks, like the case of the Internet, the difference is obvious (Fig. S9).

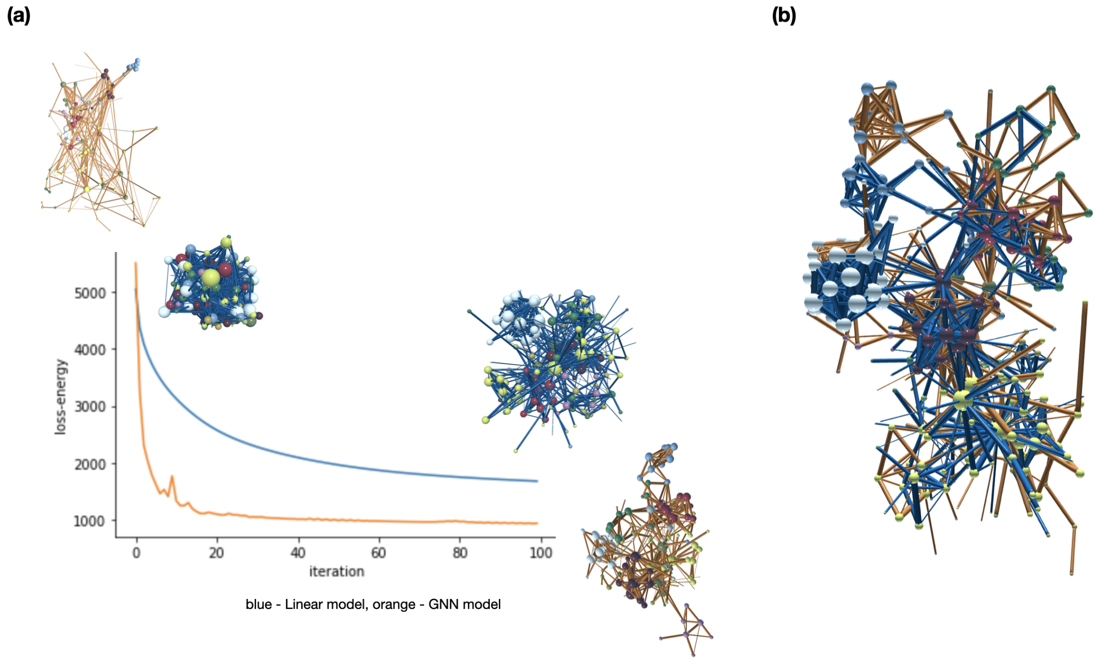

FIG. S5: **Flavor network's layout evolution.** (a) Comparing the first 100 iteration steps of the optimization process (PyTorch: RMSProp optimization) of the Linear (blue) and the GCN (orange) model. We can see that the GCN layer from the first iteration step rearranged the random node positions according to network structure and this lead to a large energy drop. (b) The two final layouts of the network on top of each other. The backbone structure is the same only the small side group positions are different. They probably are trapped in different local minima of the energy landscape.

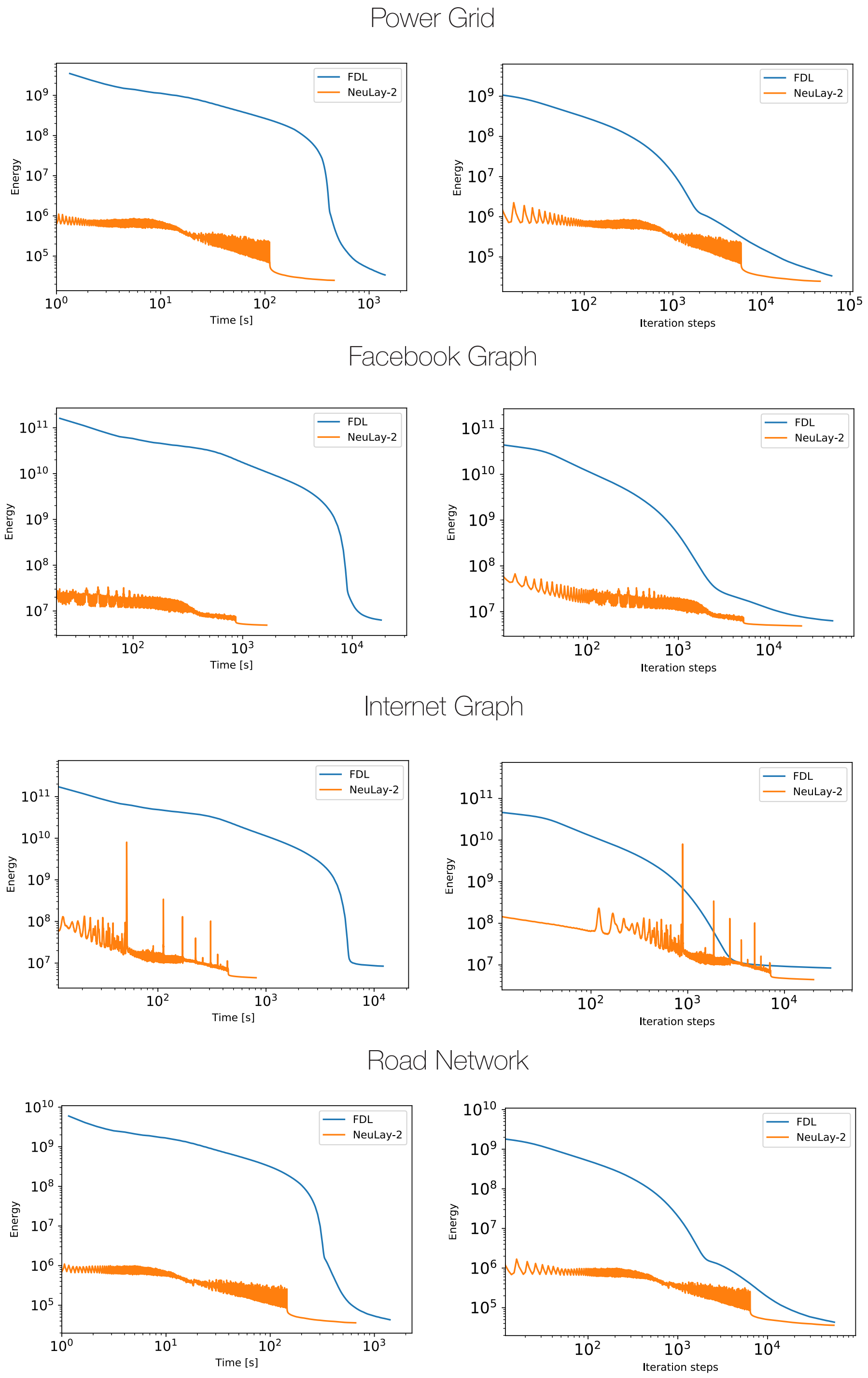

FIG. S6: **Real networks' loss function**. We show the loss curve of FDL (blue) and NeuLay-2 (orange) for the power grid, the road network, Facebook, and the Internet network, indicating that in each case NeuLay-2 converges faster, and often to much lower energies. We applied the same stopping criteria in all cases.

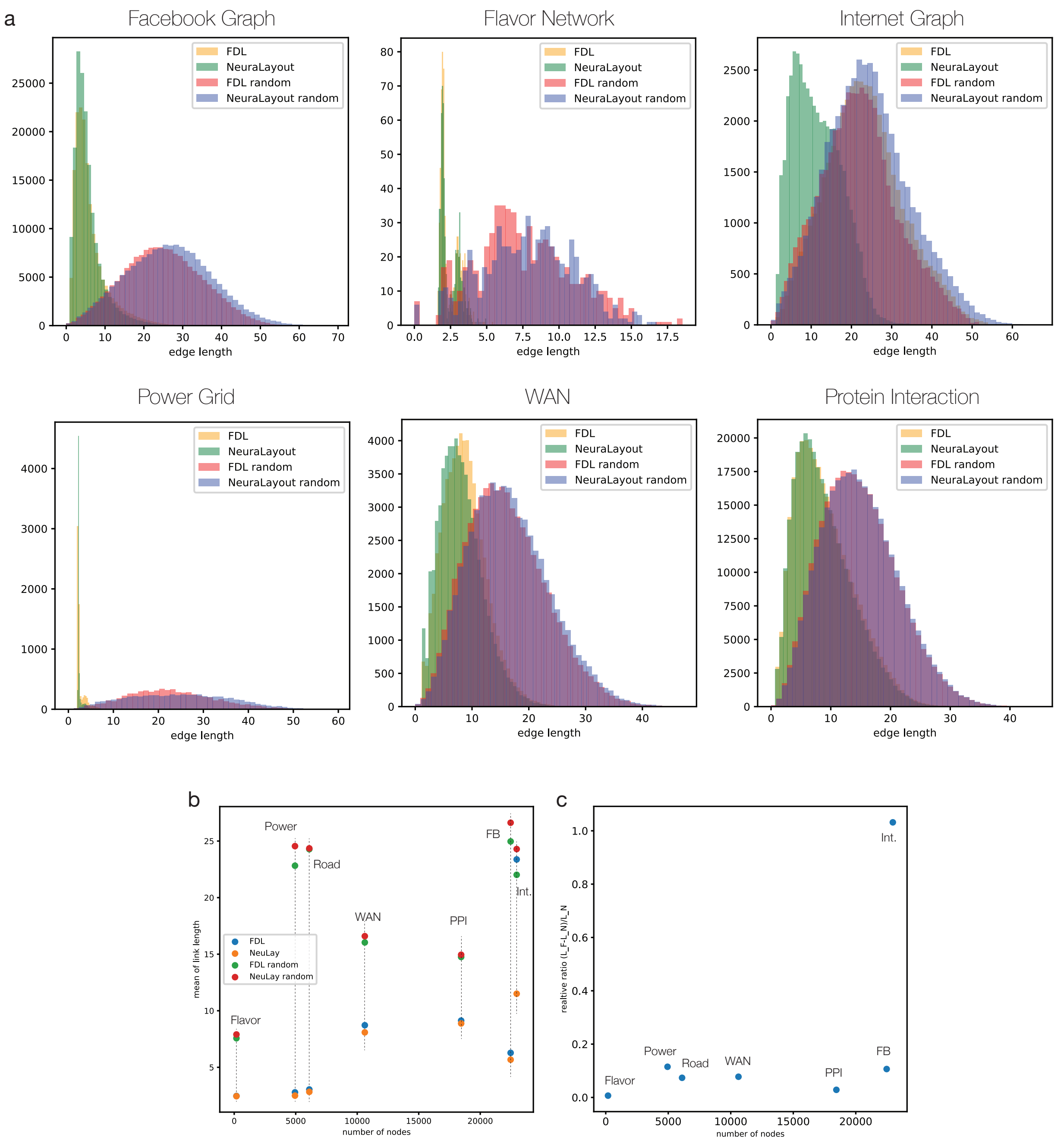

FIG. S7: **Link length distribution of real network layouts.** a) The plots show the link length distributions in real networks' layout. The green distribution is the NeuLay-2, the yellow is FDL, the red and blue are geometry preserving randomization networks. All cases the green distribution has the most short links. b) Mean link length for each network. c) Relative ratio between the mean link length of FDL and NeuLay-2 layouts. The Internet graph shows an outlier behavior, where FDL cannot find the low energy configuration of nodes.

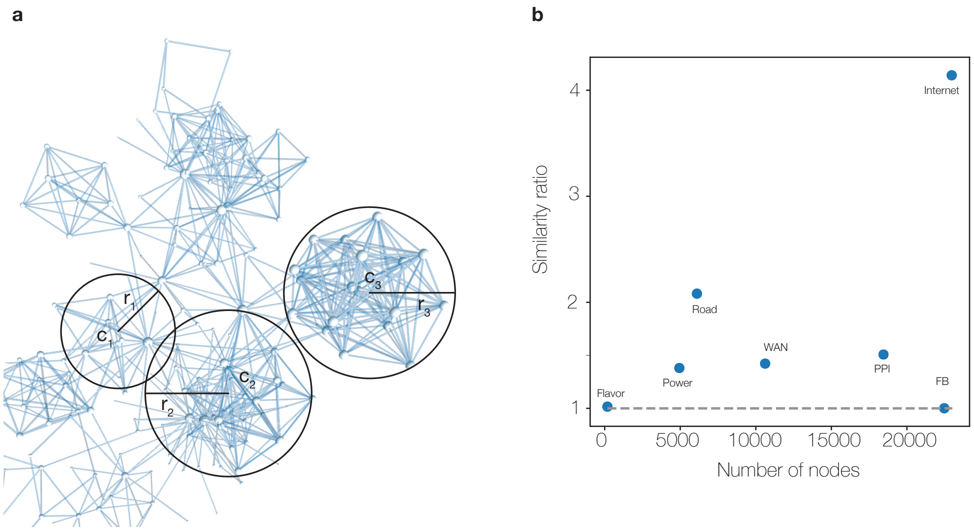

FIG. S8: **Capturing the community overlapping in real-world networks.** a) To quantify the spatial overlapping of communities in a given layout, we introduced a similarity metric, $S_{ij} = |c_i - c_j|/(r_i + r_j)$, where c_i is the center of mass of community i and r_i is the spread of community i. The spread of a community is calculated by the standard deviation of the community from the center of mass. We used the Louvain algorithm to identify the community structure of real-world networks. b) The average similarity ratio, $\sum_{i,j} S_{ij}^{NeuLay-2} / \sum_{i,j} S_{ij}^{FDL}$, of the NeuLay-2 and FDL layout for several real networks.

To quantify the visible differences between layouts generated by FDL and NeuLay-2, we measured the link length distributions and the spatial overlapping of communities (Fig. S8 b) in the layouts. Fig S7a shows the link length distributions for real networks, indicating that the NeuLay-2 algorithm always finds a layout with shorter links. Also, we find that the mean link length captures the differences between the layouts (Fig. S7 b, c). The difference is small for most real networks, with exception of the Internet. To understand the global structure of the FDL layout of the Internet, we applied the Louvain community detection algorithm to identify the community structure of the Internet graph. As we see in Fig. S9, FDL is unable to capture the local structure of the network, as the nodes from the same communities are placed far from each other. Figure S10 shows that the FDL layout communities contains more long links than the NeuLay-2, confirming the relevance of the final energy state difference for the final layout. Finally, we introduced a spatial similarity metric to measure the spatial overlap of communities for a given layout (Fig. S8 b). Spatial similarity is larger than one of the two communities that are spatially separated without any overlap. Figure S8b shows the similarity ratio for real networks, confirming that FDL successfully lays out communities in the flavor network, road network and Facebook, but it

fails for the Power Grid, WAN, PPI and the Internet.

We performed the same experiment presented in Fig. 1h using GPU to evaluate the performance of our code on different hardware. As shwon in Fig. S11 NeuLay with GCN [3], Graph Attention (GAT) [6] and Graph Network (GN) [7] GNN architecuteres exhibit a significant speedup across various network types.

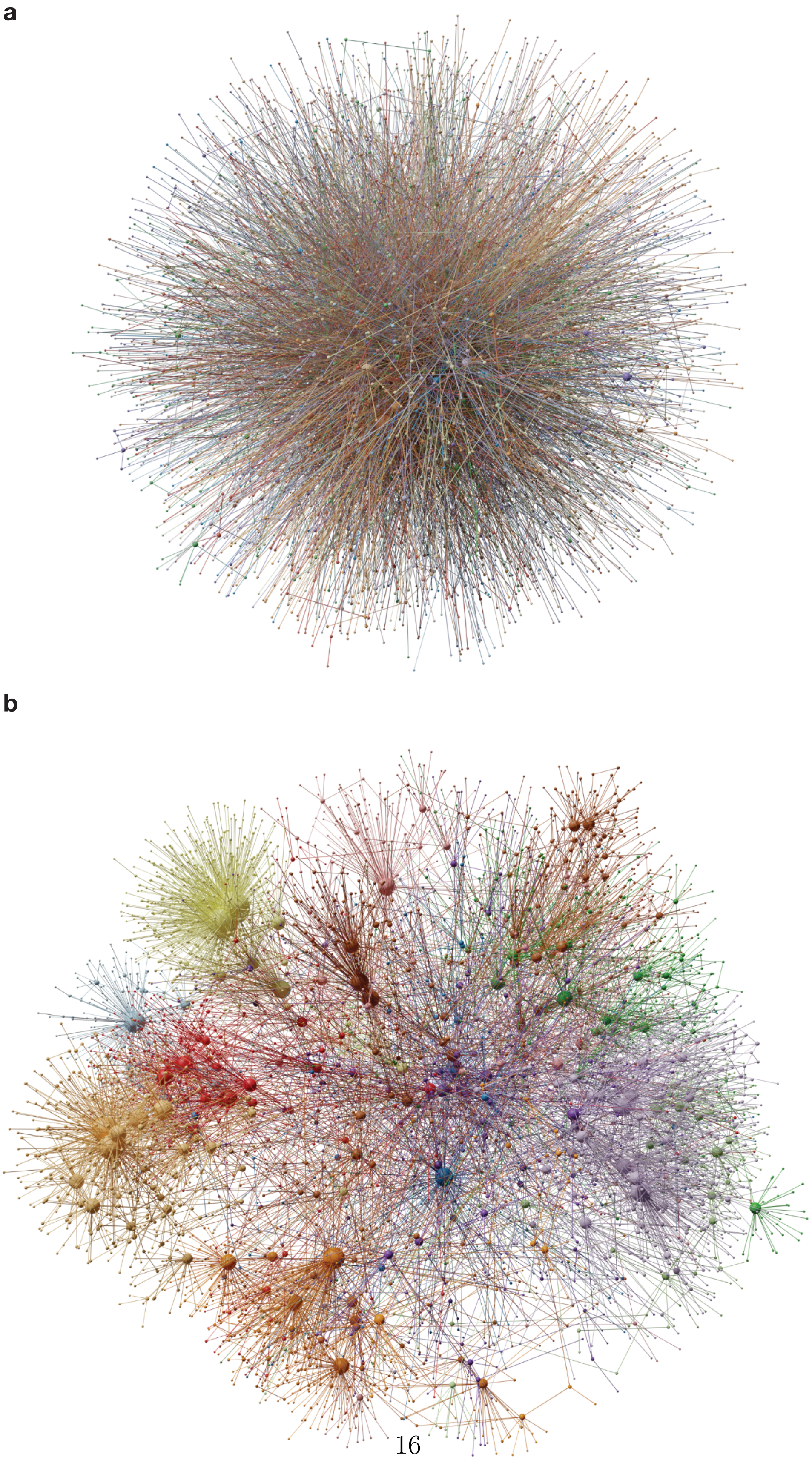

FIG. S9: **The Internet community structure.** 12 communities in the Internet layout generated by FDL **(a)** and by the NeuLay-2 **(b)**. We used Louvain algorithm to identify the community structure of the network and we highlight 12 communities in color.

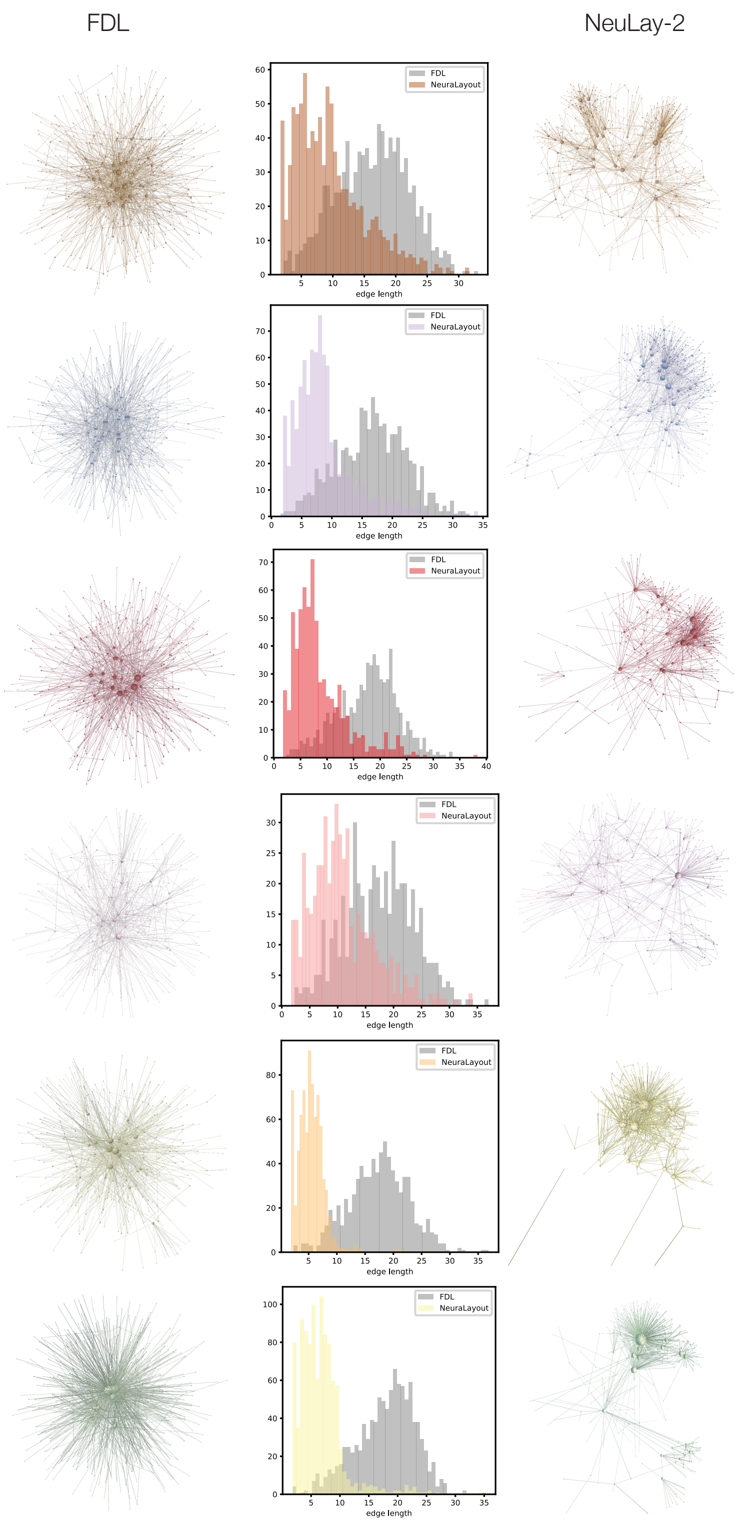
FDL
NeuLay-2
FDL
NeuraLayout
edge length

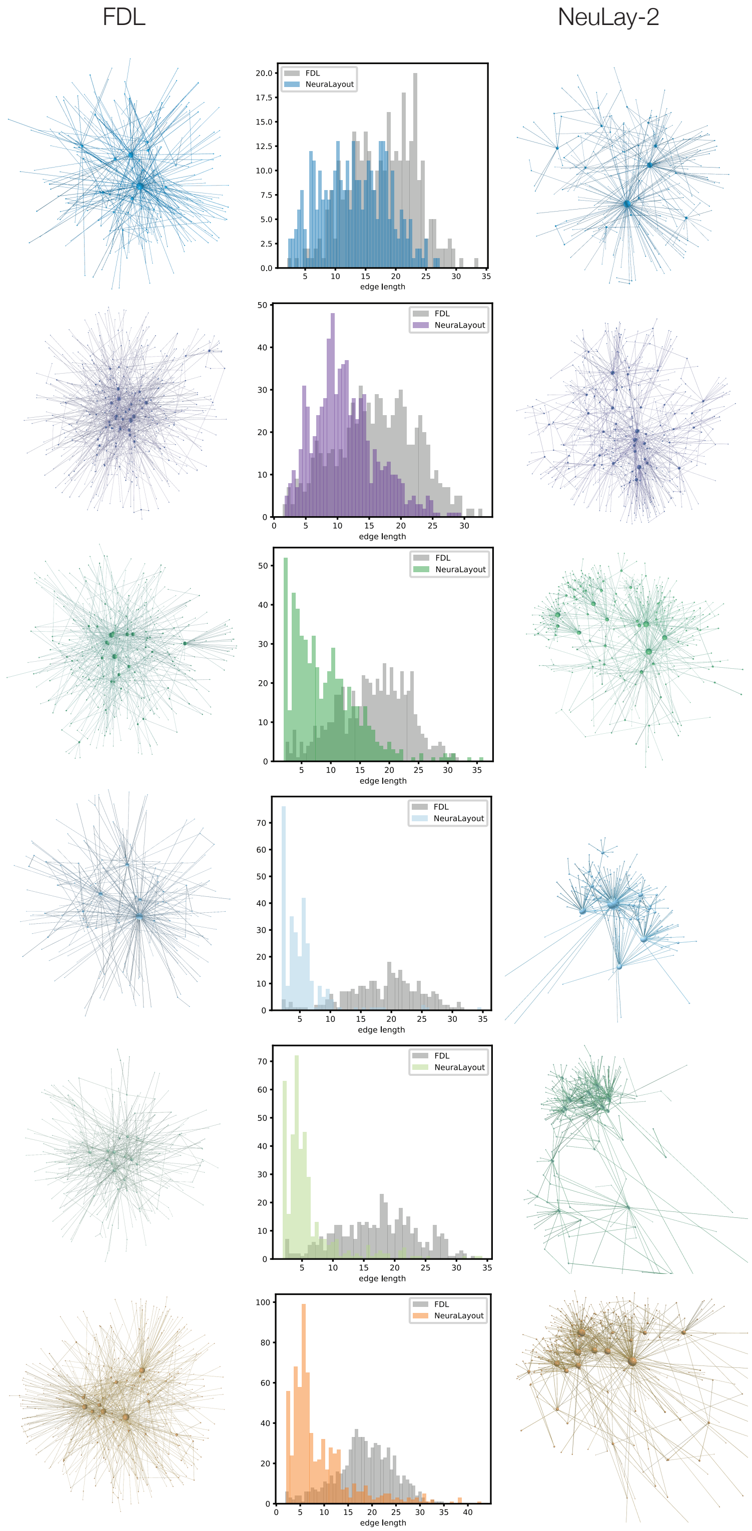

FIG. S10: **Link length distribution in communities of the Internet.** The histograms show the link length distribution in each community. On the left side of the histogram, we can find the FDL layouts, while on the right side, the NeuLay-2 one, highlighting communities in different colors

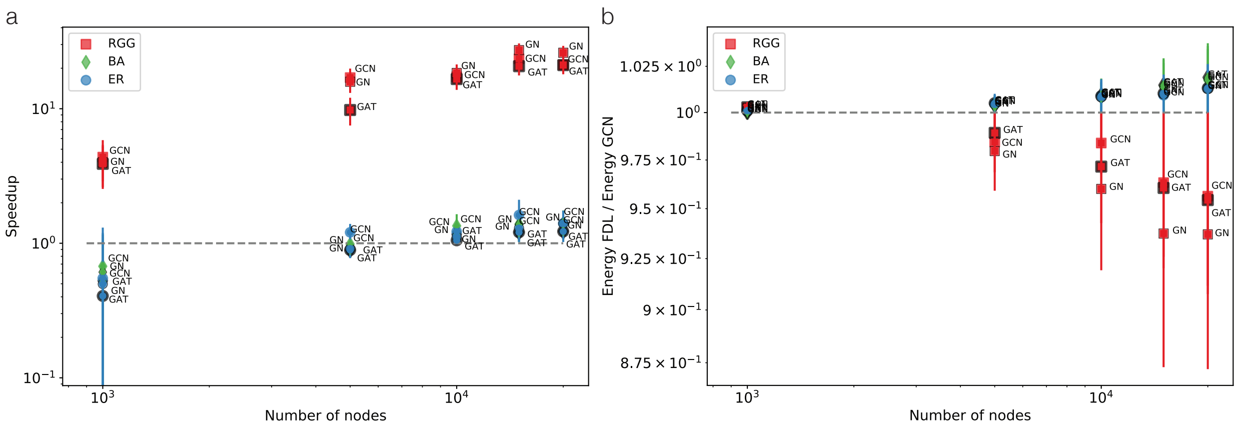

FIG. S11: **Using different graph neural networks (GCN, GAT, GN) on various network topologies.** We run all simulations on GPU hardware. Figure a) shows the speedup (FDL wall clock running time / GNN wall clock running time). GN and GCN reparametrization is slightly faster than the GAT model, yet all three models outperform the FDL algorithm. Figure b shows the various layouts' final relative energy compared to the FDL layouts' energy.

Supplementary References

1. Hoyer, S., Sohl-Dickstein, J. & Greydanus, S. Neural reparameterization improves structural optimization. *arXiv preprint arXiv:1909.04240* (2019).

2. De Bruin, T., Kober, J., Tuyls, K. & Babuška, R. Integrating state representation learning into deep reinforcement learning. *IEEE Robotics and Automation Letters* **3,** 1394–1401 (2018).

3. Kipf, T. N. & Welling, M. Semi-supervised classification with graph convolutional networks. *arXiv preprint arXiv:1609.02907* (2016).

4. Bentley, J. L. Multidimensional binary search trees used for associative searching. *Communications of the ACM* **18,** 509–517 (1975).

5. Gove, R. It pays to be lazy: Reusing force approximations to compute better graph layouts faster (2018).

6. Veličković, P., Cucurull, G., Casanova, A., Romero, A., Lio, P. & Bengio, Y. Graph attention networks. *arXiv preprint arXiv:1710.10903* (2017).

7. Battaglia, P. W., Hamrick, J. B., Bapst, V., Sanchez-Gonzalez, A., Zambaldi, V., Malinowski, M., Tacchetti, A., Raposo, D., Santoro, A., Faulkner, R., *et al.* Relational inductive biases, deep learning, and graph networks. *arXiv preprint arXiv:1806.01261* (2018).
